# METFORMIN AS A POTENTIAL PHARMACOLOGIC INTERVENTION TO IMPROVE HEALTHSPAN

**DOI:** 10.1093/geroni/igae098.0450

**Published:** 2024-12-31

**Authors:** Sara Espinoza

**Affiliations:** Cedars-Sinai Medical Center, Los Angeles, California, United States

## Abstract

Metformin is the most widely used oral antidiabetic drug, generally recommended as first-line treatment for type 2 diabetes as well as for diabetes prevention. Metformin extends lifespan in preclinical models, and several observational studies in humans suggest that metformin reduces cancer, cardiovascular disease, dementia, frailty, and all-cause mortality. Therefore, given that it is well-tolerated, safe, and widely used, there is high interest in testing its role as a potential agent to reduce age-related diseases and geriatric syndromes. The exact mechanisms by which metformin exerts these benefits are still being elucidated, but in addition to activating AMPK emerging evidence demonstrates metformin modulates several hallmarks of aging, such as epigenetic modifications, mitochondrial function, cellular senescence and the gut microbiome. Thus, metformin has pleiotropic effects which may contribute, perhaps collectively, to its aging-modulating properties. To date there are few clinical trials of metformin to directly examine metformin’s effects on human healthspan. We are currently conducting a randomized placebo-controlled clinical trial of metformin to prevent frailty in older adults with glucose intolerance, which will also examine the effects of metformin on several hallmarks of aging. Metformin is also being examined in a separate ongoing randomized placebo-controlled trial as a potential therapy to prevent Alzheimer’s disease in older adults without diabetes (including those who have prediabetes). Given its tolerability and safety profile, metformin could be quickly translated to clinical use if ongoing and future clinical trials demonstrate its efficacy in promoting healthspan.

